# Music therapy modulates mothers’ perception of their preterm infants

**DOI:** 10.3389/fpsyg.2023.1231741

**Published:** 2023-10-19

**Authors:** Susann Kobus, Marlis Diezel, Monia Vanessa Dewan, Britta Huening, Anne-Kathrin Dathe, Peter B. Marschik, Ursula Felderhoff-Mueser, Nora Bruns

**Affiliations:** ^1^Department of Paediatrics I, University Hospital, University of Duisburg-Essen, Essen, Germany; ^2^Faculty of Medicine, Centre for Translational Neuro- and Behavioural Sciences (C-TNBS), University Duisburg-Essen, Essen, Germany; ^3^Center of Artistic Therapy, University Medicine Essen, Essen, Germany; ^4^Department of Health and Nursing, Occupational Therapy, Ernst-Abbe-University of Applied Sciences, Jena, Germany; ^5^Child and Adolescent Psychiatry and Psychotherapy, Systemic Ethology and Developmental Science, University Medical Center Göttingen and Leibniz ScienceCampus Primate Cognition, Göttingen, Germany; ^6^iDN - Interdisciplinary Developmental Neuroscience, Division of Phoniatrics, Medical University of Graz, Graz, Austria; ^7^Center of Neurodevelopmental Disorders (KIND), Center for Psychiatry Research, Department of Women’s and Children’s Health, Karolinska Institutet, Stockholm, Sweden

**Keywords:** music therapy, neonatology and pediatric intensive care, neonatology, music, preterm infants, premature infants, neonatal intensive care unit

## Abstract

Premature birth imposes considerable challenges on the preterm infant, but also challenges the mother, who may not yet be prepared for motherhood and encounter psychological stress during the post-partum period. This secondary analysis of a prospective randomized controlled trial (RCT) aimed to investigate how mothers perceive their preterm infants. We surveyed 33 mothers of preterm infants born before 32  weeks of gestation, who were participating in an RCT on music therapy. We employed the neonatal perception inventory (NPI), an instrument designed to assess the mother’s perception and expectations regarding her infant immediately after birth. Infants were randomly assigned to either standard care (control group) or standard care plus music therapy (intervention group). Eighteen mothers from the intervention group participated in the survey (mean age 34.1  ±  4.6  years) and 15 mothers from the control group (mean age 29.6  ±  4.2  years). At the time of the infant’s hospital discharge, mothers rated their expectations of how they felt a baby should behave (NPI I) and how they perceived their own infant (NPI II). The NPI score difference was calculated by subtracting the NPI II from the NPI I. Mean NPI I scores were comparable between both groups, but NPI II scores in the intervention group were better [18.0, 95% confidence interval (CI) 16.6–19.4] than in the control group (19.1, 95% CI 18.0–20.3). The relative probability of mothers rating their own baby as better than average was 1.94 (95% CI 1.00–3.79) for mothers whose infants received music therapy. These findings suggest that music therapy in the neonatal intensive care unit can positively influence mothers’ perception of their hospitalized preterm infant.

## 1. Introduction

The transition to motherhood, marking the onset of the mother–child relationship, represents a crucial phase of adjustment within a family, entailing important changes in a mothers lifestyle and daily routines (Price et al., 2000; Deave et al., 2008). This transition and adaptation to the new role can pose substantial challenges, even for full-term infants’ mothers. However, preterm birth can exacerbate these challenges: Mothers of preterm infants are at increased risk of experiencing negative emotions toward their baby in the first few months compared to term infants’ mothers, possibly due to reduced early contact with their infant (Wolf et al., 2002; Henderson et al., 2016). They frequently perceive their baby as more difficult than the average infant and use more negative adjectives to describe their baby. At the same time, it is well-established that a mother’s postnatal perception of the infant and the quality of the mother-infant bond play a major role in the well-being of mothers and their newborns (Smorti et al., 2020; Ahmadinezhad et al., 2022). While a positive maternal perception during the first months of life does not completely prevent developmental difficulties of the infant, the absence of a positive maternal perception of a newborn is associated with emotional development issues in the child (Broussard, 1997).

Typically, premature infants are immediately separated from their mothers after birth to ensure optimal medical care (Ncube et al., 2016). In today’s neonatal intensive care units (NICUs), the challenge is to provide the best possible medical developmental care for a preterm infant and at the same time to support mothers in caring for and bonding with their infant (Tilokskulchai et al., 2002; Aagaard and Hall, 2008). It is undeniable that mothers of extremely preterm infants experience a higher level of psychological distress during the period immediately after the birth than mothers of full-term babies (Holditch-Davis et al., 2003). However, the adaptation to the premature mother’s role can be promoted by empowering mothers to cope with negative experiences and emotions, especially in the NICU (Heydarpour et al., 2016).

Therefore, it is important to identify therapeutic interventions aimed at alleviating possible stress symptoms in the infants (Kobus et al., 2021a) and their mothers, thereby promoting a positive mother–child relationship (Benzies et al., 2013). Research has shown that targeted interventions such as music therapy can help and support mothers of preterm infants to establish positive parenting routines that are beneficial for themselves and their babies (Lau et al., 2020). Music therapy is an individualized and family-integrated therapy approach (Haslbeck and Bassler, 2020), offering various benefits for preterm infants and their parents (Ettenberger et al., 2016). By including the parents in the music therapy sessions, they can establish closer contact with their infants (Haslbeck, 2014; Ettenberger et al., 2016). Furthermore, music therapy serves as a stress-reducing intervention capable of not only temporary stabilization of a preterm infant (Standley, 2012), but exerting positive effects on the mother’s behavior and emotions (Kobus et al., 2022c). Music therapy in the NICU reduces symptoms of anxiety and depression in couples during their NICU stay (Kehl et al., 2020). Parents perceive music therapy as a positive, supportive resource for themselves and their infants, which alleviates challenges faced the families during the difficult early days in a NICU (van Dokkum et al., 2020; Kobus et al., 2021b).

However, it remains unknown whether music therapy in preterm infants has an impact on how mothers perceive their infants. The aim of our study was to investigate the effect of live music therapy in the NICU, provided from the second week of life until hospital discharge, on mothers’ perception of their preterm infants. We used the neonatal perception inventory (NPI) to assess how mothers of infants born <32 gestational weeks perceived their own infant compared to their expectation of an average baby at the time of hospital discharge. These analyses expand the understanding of music therapy for preterm infants on maternal stress levels, building upon previously published research from the same group of mother-infant pairs, which showed that music therapy reduces depressive symptoms and distress in mothers during their infants’ NICU stay (Kobus et al., 2022c).

## 2. Methods

### 2.1. Study design

Mothers of preterm infants born before 32 weeks gestational age at the neonatal intensive care unit of the University Hospital Essen (Germany) between January 2020 and May 2021 who were participating in a randomized controlled trial (RCT) on music therapy in preterm infants filled out the Neonatal Perception Inventory (NPI) at the infants’ discharge from hospital. Fifteen months after the beginning of a randomized controlled trial on the effect of music therapy on preterm infants’ short- and long-term outcomes (German Clinical Trials Registry number: DRKS00025753), the survey was added to the study protocol and mothers of all subsequently included infants were prospectively surveyed. A previous study using the same cohort of mothers and infants investigated the impact of music therapy on maternal distress as measured by Center for Epidemiologic Studies Depression Scale (CES-D) and Impact of Events Scale-Revised (IES-R) (Kobus et al., 2022c). The present study examines the effect of music therapy on mothers’ perception of their infant at hospital discharge, a parameter that is a correlate of mother-infant connectedness (Holditch-Davis et al., 2007; Nicolaou et al., 2009).

### 2.2. Infant eligibility and recruitment for the randomized controlled trial

Infants born between January 2020 and May 2021 at the University Hospital Essen <32 weeks gestational age without congenital hearing disorders, intraventricular hemorrhage grade III after Papile, periventricular infarction or cerebral malformations were eligible for participation in the main study (Kobus et al., 2022b). The declaration of consent was signed by the parents at a minimum age of 72 h and a maximum age of 7 days of their infant. The randomized controlled trial was approved by the local ethics committee of the medical faculty of the University of Duisburg-Essen (18-8035-BO).

### 2.3. Randomization and music therapy intervention

In the main study, eighty clinically stable preterm infants were recruited and randomized. Randomization was carried out by one of the investigators (MD, MVD, NB, SK) immediately after inclusion into the trial by opening a numbered sealed envelope that contained information about the randomization group (standard care or standard care plus music therapy). Prior to the start of the study, a randomization list (allocation 1:1, block randomization with blocks of 10) was created and all envelopes prepared.

Forty stable infants of the intervention group received individually live-performed music therapy from a qualified music therapist in addition to standard medical care twice per week until their hospital discharge (Kobus et al., 2021a, 2022c). During a session with a duration of about 10–15 min, the child was in the incubator, in a warming bed or had direct physical contact with the parents. Based on the reactions of the infant and synchronized with the respiratory pattern, the music therapist sang improvised melodies or played soft tones with the instrument Sansula throughout the entire session. The Sansula is an instrument which creates an enveloping soft sound. The tones were played at low and soft volume and the tempo adjusted to the breathing rhythm of each individual infant. The faster the breathing rhythm, the lower the tempo frequency of the tones so that the infants listened to calm music. The music was improvised individually for each infant. During the intervention, infants remained in the position they had been in before the session (incubator, heating bed, bed or on their mother’s chest/arms). At the beginning of the music therapy session the therapist sat or stood next to the infant and began to play or sing a note. This note sounded for about 3 s, followed by a silence of about 3 s. The next tone then sounded at a different tone level. The periods of silence were gradually reduced within the first 5 min. Depending on the infants breathing rhythm, up to 30 tones per minute were played. If the breathing rhythm was high, the tones were synchronized with every second or third breath to calm down the infant. Usually, after a maximum of three tones there was a pause corresponding to the length of one or two tones. During the last 5 min of the session, the tempo was reduced again, and the breaks were lengthened. The pitch lowered at the end and the final note was a deep tone. Parents received the delivered music without active participation. All clinical data of the preterm infants and their mothers were analyzed and the mothers’ presence during an intervention was documented.

### 2.4. Neonatal perception inventory

The Neonatal Perception Inventory (NPI) was developed by Broussard and Hartner to measure mothers’ perception of their own neonate compared to their expectations of an average baby shortly after birth (Povedano et al., 2011). The NPI assesses six categories (crying, feeding, spitting, elimination, sleeping and predictability) on a five-point Likert scale, with 5 being the worst and 1 the best score. These items reflect the functioning of the mother-infant dyad during the neonatal period (Broussard and Hartner, 1971) and mothers provide their own baseline data by rating their own infants (NPI II Score) and a hypothetical average baby (NPI I score) (Broussard and Hartner, 1970; Povedano et al., 2011). The NPI II Score (own baby) is then subtracted from the NPI I score (average baby) to form the total score (NPI score difference).

The NPI sores I and II provide the parents perception about their own infants (NPI II Score) and a hypothetical average baby (NPI I score). It is important to assess not only the difference but also the absolute values, as the intervention might have modulated not only the perception of the own baby but also the expectation. The NPI score difference reflects the risk of later socioemotional problems in childhood, whereas the NPI II score was the most stable in a validation study (Camp, 1982).

An NPI score difference below zero implies a negative perception (negative direction), in which the mothers perceive their own infant to be more difficult than the average baby. A difference greater than zero (positive direction) implies a positive perception of their own infant being less difficult than the average baby, which is associated with a low risk of later socioemotional problems in childhood (Palisin, 1981).

The five-point Likert scale of NPI has been validated, with the NPI II proving most robust (Camp, 1982).

### 2.5. Outcomes

The primary outcomes were mothers’ mean NPI scores and mean score differences at the infant’s hospital discharge in the intervention and control group and the difference between groups. Secondary outcomes were the proportion of own babies rated better than the expected average and the relative “risk” (= probability) of rating the own baby better than average in each group. Additionally, mean values for subscores of the NPI were calculated.

### 2.6. Statistical analyses

Categorical variables are presented as counts and relative frequencies. Continuous variables are presented as mean and standard deviation (SD) or 95% confidence intervals (CI) if evenly distributed and as median with interquartile range (IQR) if skewed. We analyzed the mothers’ NPI I and II total scores, score differences between NPI I and NPI II and the scores of the six sub-categories crying, feeding, spitting, elimination, sleeping and predictability. For the main outcomes (NPI I and II and NPI score difference), mean and 95% CIs were calculated. To adjust for differences in NPI I, an analysis of covariance was carried out as a sensitivity analysis using a generalized linear model.

Effect sizes were calculated according to Cohen [Cohen’s *d* = (*M*_2_ − *M*_1_)/*SD*_pooled_] in order to provide an objective estimation of the strength of effect in addition to the crude and adjusted means. The relative “risk” (= probability) of having a positive direction in the perception of the own baby compared to average babies between the two groups was calculated using an unadjusted linear model.

SAS Enterprise Guide 8.4 (SAS Institute Inc., Cary, NC, United States) was used to perform statistical analyses and produce figures.

## 3. Results

### 3.1. Participants

**Figure 1:**
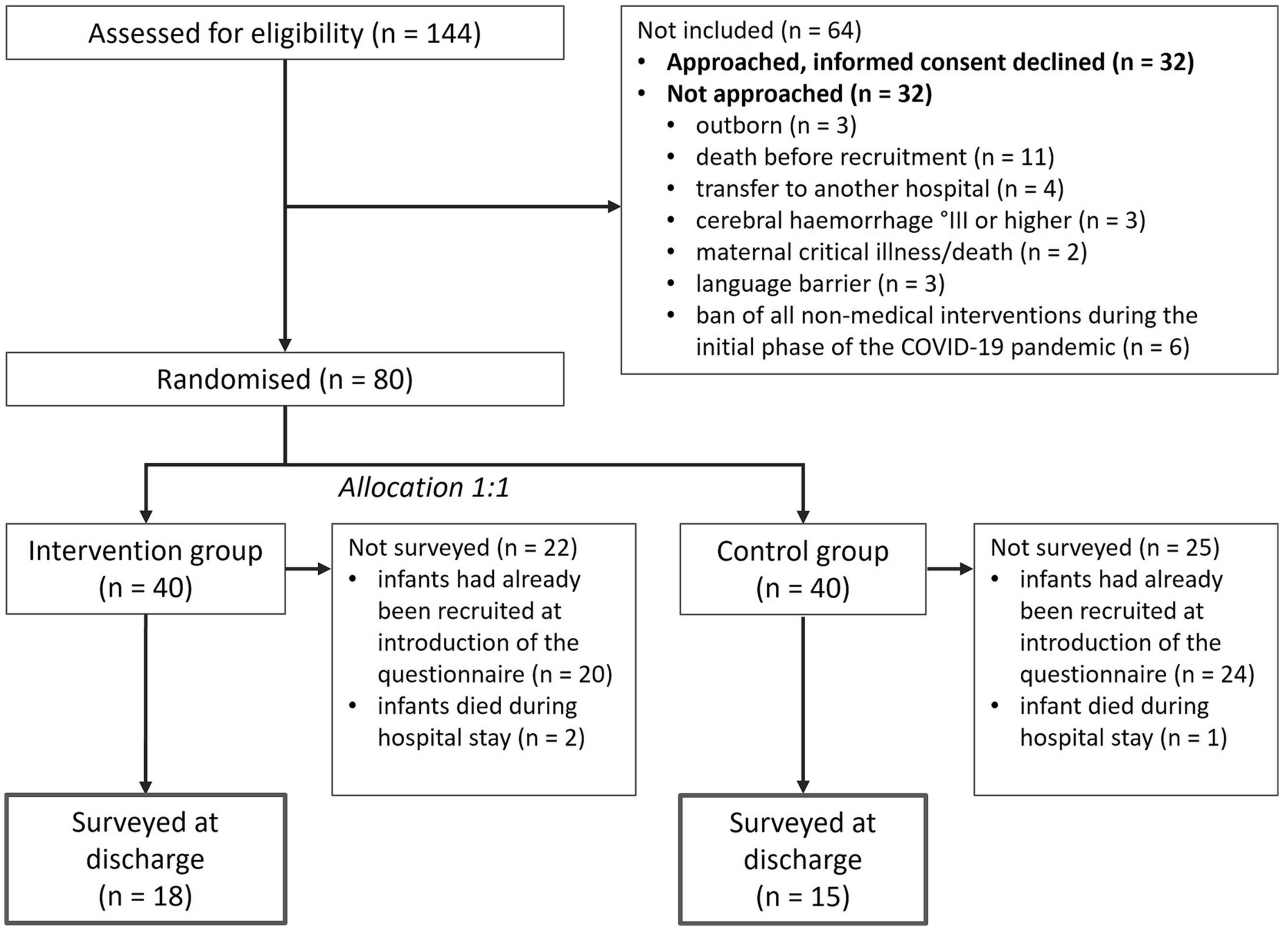
Flow chart of the included and excluded participants of the study.

During the period of the main study from October 2018 to May 2021, 144 premature infants born at <32 gestational weeks were screened for eligibility (Figure 1). Eighty infants were randomized to either the intervention or the control group.

The NPI questionnaire was added to the main study beginning at the 45th of 80 infants, leading to 36 infants’ mothers to potentially be included in the NPI analysis. Because three infants died before discharge (Figure 1), 33 infants (Table 1) and their mothers (Table 2) were included for the NPI analysis. The infants of the control group had longer median hospital stays, in spite of similar gestational ages and baseline characteristics at birth (Table 1).

**Table 1:** Clinical characteristics of the included patients in the intervention and the control group.

	Intervention group (*n* = 18)	Control group (*n* = 15)
Male, n (%)	8 (44%)	12 (80%)
GA (weeks), mean ± SD (range)	29 + 1 ± 2.4 (23 + 6–31 + 5)	28 + 5 ± 2.5 (22 + 6–31 + 5)
Birth weight (g), mean ± SD (range)	1,273 ± 390 (590–1790)	1,094 ± 368 (545–1,800)
Birth weight < 10th Percentile, n (%)	0 (0%)	3 (20%)
Body length at birth (cm), mean ± SD (range)	37.7 ± 4.4 (30.0–45.0)	36.6 ± 4.9 (29.0–46.0)
Head circumference at birth (cm), mean ± SD (range)	26.8 ± 2.9 (21.0–30.5)	26.1 ± 2.6(21.0–31.5)
Apgar score at 10 min, median (IQR)	9 (8–9)	9 (7–9)
Parenteral nutrition (days), median (IQR)	9 (7–10)	10 (7–15)
Early onset sepsis, n (%)	2 (11%)	2 (13%)
Late onset sepsis, n (%)	4 (22%)	5 (33%)
Corrected GA at discharge (weeks), mean ± SD (range)	38 + 1 ± 2.7 (34 + 3–42.3)	40 + 2 ± 2.6 (35 + 1–45 + 1)
Weight at discharge (g), mean ± SD (range)	2,758 ± 512 (2,085–3,780)	3,419 ± 827 (2,400–5,290)
Body length at discharge (cm), mean ± SD (range)	47.5 ± 3.0 (42.3–53.0)	49.8 ± 3.7 (43.0–56.0)
Head circumference at discharge (cm), mean ± SD (range)	32.7 ± 1.7 (30.0–35.5)	35.0 ± 2.4 (31.0–38.5)
Respiratory support at discharge, n (%)	0 (0%)	0 (0%)
Nasogastral tube at discharge, n (%)	1 (6%)	1 (7%)

GA, gestational age; SD, standard deviation; IQR, interquartile range; cm, centimeter; g, gram.

**Table 2:** Sociodemographic characteristics of the mothers in the intervention group and the control group.

	Intervention group	Control group
Mothers, n (%)	18 (55%)	15 (45%)
Age at birth (years), mean ± SD (range)	34.1 ± 4.6 (25.0–40.0)	29.6 ± 4.2 (20.0–36.0)
Gravida, mean ± SD (range)	2.9 ± 2.2 (1.0–8.0)	2.6 ± 2.0 (1.0–8.0)
Primagravida, n (%)	8 (44%)	5 (33%)
Multigravida, n (%)	10 (56%)	10 (67%)
Number of current child, mean ± SD (range)	2.4 ± 1.7 (1.0–6.0)	2.3 ± 1.9 (1.0–8.0)
Birth mode		
Primary cesarean section, n (%)	9 (50%)	7 (47%)
Secondary cesarean section, n (%)	2 (11%)	1 (7%)
Emergency cesarean section, n (%)	3 (17%)	4 (27%)
Spontaneous, n (%)	4 (22%)	3 (20%)

A total of 286 music therapy sessions were conducted in the intervention group between corrected gestational ages of 24 + 5 and 42 + 1 weeks. The mean duration of each music therapy session was 29.6 min (range 10–50 min), with parents present in 105 (37%) sessions. On average, infants received six interventions together with their mother.

### 3.2. Neonatal perception inventory

**Figure 2:**
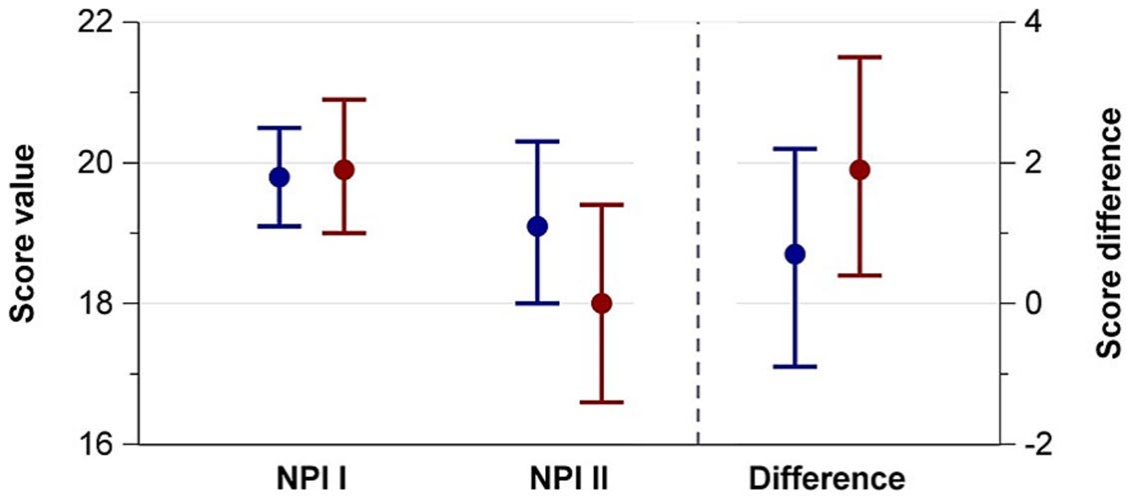
NPI and II scores and score differences at preterm infants’ hospital discharge of mothers whose infants received music therapy during their stay (red) or did not (blue).

The NPI I score did not differ between mothers whose infants received music therapy and mothers whose infants did not. Regarding the mothers’ perception of their own infant, NPI II scores in the music therapy group were lower (=better) than in the control group [18.0 (95% CI 16.6–19.4) vs. 19.1 (95% CI 18.0–20.3)] (Table 3 and Figure 2). The mean differences between NPI I and II were larger (= more positive) in mothers with music therapy for the overall score difference (Table 3) and sub-scores (Table 4 and Figure 2).

**Table 3:** Crude and adjusted mean NPI scores and score differences in the intervention and control group at the infants’ discharge from hospital.

	Mean (95% CI)			Adjusted mean (95% CI)	
	Intervention group	Control group	Effect size*	Intervention group	Control group
NPI I score (average babies)	19.9 (19.0–20.9)	19.8 (19.1–20.5)	0.04	–	–
NPI II score (own baby)	18.0 (16.6–19.4)	19.1 (18.0–20.3)	0.30	17.7 (15.7–19.7)	19.2 (17.0–21.4)
NPI score difference	1.9 (0.4–3.5)	0.7 (−0.9–2.2)	0.27	1.9 (−0.1–3.9)	0.4 (−1.8–2.6)

CI, confidence interval. *Cohen’s d between intervention and control group.

**Table 4:** Sub-scores of the NPI I and II scores by intervention.

					Difference between average and own baby	
	Intervention group		Control group		Intervention group	Control group
	Average babies	Own baby	Average babies	Own baby		
Crying	3.4 (3.2–3.7)	2.4 (2.0–2.9)	3.5 (3.2–3.9)	2.9 (2.5–3.3)	1.0 (0.6–1.4)	0.7 (0.1–1.2)
Feeding	3.1 (2.7–3.4)	3.3 (2.9–3.8)	2.7 (2.4–3.1)	3.0 (2.4–3.6)	−0.3 (−0.7–0.2)	−0.3 (−0.9–0.3)
Spitting or vomiting	3.5 (3.1–3.9)	3.0 (2.5–3.5)	3.3 (3.1–3.6)	2.9 (2.4–3.3)	0.5 (−0.1–1.1)	0.5 (0.0–0.9)
Sleeping	3.0 (2.6–3.4)	2.2 (1.8–2.6)	3.1 (2.8–3.4)	3.3 (2.9–3.7)	0.8 (0.2–1.3)	−0.1 (−0.6–0.4)
Eliminating	3.5 (3.0–4.0)	3.7 (3.3–4.0)	3.5 (3.1–3.8)	3.7 (3.2–4.2)	−0.2 (−0.7–0.4)	−0.3 (−1.0–0.5)
Predictability	3.2 (2.7–3.6)	3.1 (2.4–3.7)	3.3 (3.0–3.7)	3.5 (3.0–4.0)	0.1 (−0.5–0.8)	−0.1 (−0.8–0.5)

All data represent mean and 95 confidence intervals.

The proportion of mothers who rated their baby better than average was higher in the intervention group for the overall score difference (78% vs. 40%) (Table 5). The relative probability of rating the own baby better than average was 1.94 (95% CI 1.00–3.79) in the intervention group.

**Table 5:** Comparison of NPI direction between mothers of preterm infants with and without music therapy.

	Intervention group (*n* = 18)	Control group (*n* = 15)
Positive direction (better than average)	14 (78%)	6 (40%)
Negative direction (less than average)	4 (22%)	8 (53%)
No direction (average)	0 (0%)	1 (7%)

Of the NPI sub-scores, the largest differences between the intervention and non-intervention groups were observed for crying and sleeping [mean difference for crying 1.0 (95% CI 0.6–1.4) and mean difference for sleeping 0.8 (95% CI 0.2–1.3)] (Table 4).

## 4. Discussion

In this RCT, mothers of preterm infants who received live music therapy in the NICU rated their own infant more favorably on the NPI compared to mothers whose infants did not receive music therapy. The overall NPI scores in the intervention group were more positive compared to the control group, with small to medium effect sizes for both the total NPI II scores and the NPI score difference.

Although music therapy in the NICU is aimed primarily at preterm infants, a growing evidence suggests beneficial effects not only on the infants’ well-being but also on their parents (Palisin, 1981; Loewy et al., 2013; Bieleninik et al., 2016; Kobus et al., 2021a, 2022a,c; Menke et al., 2021). While our previous investigations on music therapy with preterm infants have focused on directly measuring parental well-being or assessing the perception of the music therapy intervention (Kobus et al., 2021b, 2022c), this study marks the first attempt to evaluate the impact of music therapy on mothers’ perception of their own infant in the NICU. Our findings indicate that the mothers’ expectations of an average baby did not differ between the intervention and control group. However, there was a more positive perception of their own infant in the intervention group. Hence, it appears that while expectation remained unaffected, the perception of their own infant was positively influenced by the music therapy intervention.

It is well-acknowledged that a mother’s expectations can exert influence on an infant’s behavior and development (Broussard and Hartner, 1970; Van den Akker et al., 2022). Notably, maternal expectations of their unborn child’s temperament appear to extend into postnatal reality, shaping early interactions between the mothers and their child that can further influence the child’s temperament development (Van den Akker et al., 2022). A positive mother-infant perception may foster the development of a more stable relationship between mother and infant (Jacobsen et al., 2014). The core concept underlying family-centered music therapy is to improve the emotional interaction between the parents and infant (Jacobsen et al., 2014). This effect has been shown in related fields in the context of music therapy and emotional interaction between parents and their child: In older children with autism spectrum disorders, music therapy improved mothers’ confidence to engage with their child and new understanding of the child’s interests and strengths (James et al., 2015; Thompson, 2017). Family-centered music therapy offers parents a novel perception of their child by experiencing them in new ways during music therapy (Annesley et al., 2020). Furthermore, music therapy encourages parents to adopt a more playful and engaged interaction with their children (Kaenampornpan, 2015). Parents link these changes to feeling a closer connection to their child, resulting in an overall improvement in the quality of their relationship (Kobus et al., 2021b). Interestingly, the results of our study align with these previous findings, even though the music intervention itself did not include active parental engagement (Kobus et al., 2021b, 2023). Consequently, it remains uncertain whether the modulation of maternal perception was directly influenced by the music intervention or if it occurred indirectly via receiving a “special” intervention.

The primary limitations of this study include a small sample size and a one-dimensional quantitative assessment of mother-infant relationship using the NPI questionnaire, without longitudinal NPI assessment. Qualitative assessment of mother-infant perceptions might uncover additional dimensions of changes to the mother-infant interaction. Another relevant constraint is that mothers were only present in 37% of the sessions. As a result, it is possible that the influence on maternal perception may be solely attributed to the music therapy intervention itself but rather to the awareness that the infant was receiving a “special” intervention. Since the primary focus of the main RCT was not designed to address this question, we can only conclude that there was a difference in maternal perception between music and no music therapy, without determining whether a different form of music therapy intervention, such as one involving active maternal engagement, would have yielded more pronounced effects on maternal perception.

In summary, this study adds evidence to the existing literature, supporting the idea that music therapy offers parents opportunities to perceive themselves and their infants or children in novel ways (Allgood, 2005; Lindenfelser et al., 2012; Thompson, 2017; Kobus et al., 2021b). Further research is needed to explore moderating impacts of music therapy on family dynamics and parent-child relationships. Decision makers within the (German) healthcare system should become more aware of the comprehensive effects that music therapy exerts not only on the patients themselves but on the entire family unit. The growing evidence of its positive psychological effects should prompt the inclusion of music therapy as a standard treatment covered by the health system, rather than requiring private funding in order to provide this family-nurturing intervention.

## 5. Conclusion

In this study, individually delivered live music therapy for preterm infants at NICU positively impacted the mother-infant relationship. This findings contributes to the growing body of evidence demonstrating that music therapy can have nurturing psychological effects, not only on patients but also their families, particularly during critical, chronic or life-shortening illness.

## Data availability statement

The original contributions presented in the study are included in the article/supplementary material, further inquiries can be directed to the corresponding author/s.

## Ethics statement

The studies involving humans were approved by the Ethik-Kommission Universität Duisburg-Essen Robert-Koch-Straße 9-11, 45147 Essen. The studies were conducted in accordance with the local legislation and institutional requirements. Written informed consent for participation in this study was provided by the participants’ legal guardians/next of kin.

## Author contributions

NB, MVD, UF-M, and SK: study design. SK and MD: delivery of music therapy. NB: statistical analyses and figures. SK: drafting of manuscript. NB, BH, MVD, PM, UF-M, and A-KD: critical revision. All authors have read and agreed to the published version of the manuscript.
